# Capsulolabral Advancement and “Suture-First” Knot-Less Technique for Chronic Posterior Glenolabral Articular Disruption Lesion of the Shoulder

**DOI:** 10.1016/j.eats.2025.103564

**Published:** 2025-05-03

**Authors:** Ranajit Panigrahi, Aruddha Sarkar

**Affiliations:** a Department of Orthopaedics, Hitech Medical College, Bhubaneswar, India; b Arthroscopy and Sports Injury, Kalinga Hospital Limited, Bhubaneswar, India

## Abstract

Glenoid labrum articular disruption (GLAD lesions) have been classically described along the anterior and inferior aspect of the glenoid. Posterior GLAD lesions are a rare variant in which the posterior-inferior glenoid and the labrum are involved. The management of chronic GLAD lesions is challenging and requires removal of loose fibrous tissue, microdrilling, and capsulolabral advancement in order to minimize an exposed articular defect and re-establish glenolabral integrity. We describe a technique of capsulolabral advancement with “suture-first” knotless anchors to manage a case of chronic posterior GLAD lesion of shoulder.

Glenoid labrum articular disruption (GLAD), first documented by Neviaser,^1^ describes combined labral pathology and adjacent articular cartilage lesion. These lesions are a well-established cause of shoulder pain, often without frank instability.^2^ They can also be source of shoulder pain in glenohumeral instability and degenerative shoulder disease.^3^, ^4^, ^5^ GLAD lesions have been classically described along the anterior and inferior aspect of the glenoid. Similar lesions involving the posteroinferior glenoid have been documented and termed as posterior GLAD lesions, wherein patients present with posterior shoulder pain with or without frank instability.^6^ Management of these lesions consists of restoring the glenoid articular surface, minimizing the exposed articular defect, and re-establishing glenolabral integrity.^7^^,^^8^ Chronic posterior GLAD lesions should be managed using chondral procedures and labral advancement to provide satisfactory clinical and functional outcomes to the patient. Here we describe a technique of capsulolabral advancement with “suture-first” knotless anchors to manage a case of chronic posterior GLAD lesion.

## Surgical Technique

### Patient Positioning

With the patient under general anesthesia and in the lateral decubitus position, the operative arm is placed in 10 lbs of suspension traction after prepping and draping in sterile condition.

### Arthroscopic Approach

**Fig 1:**
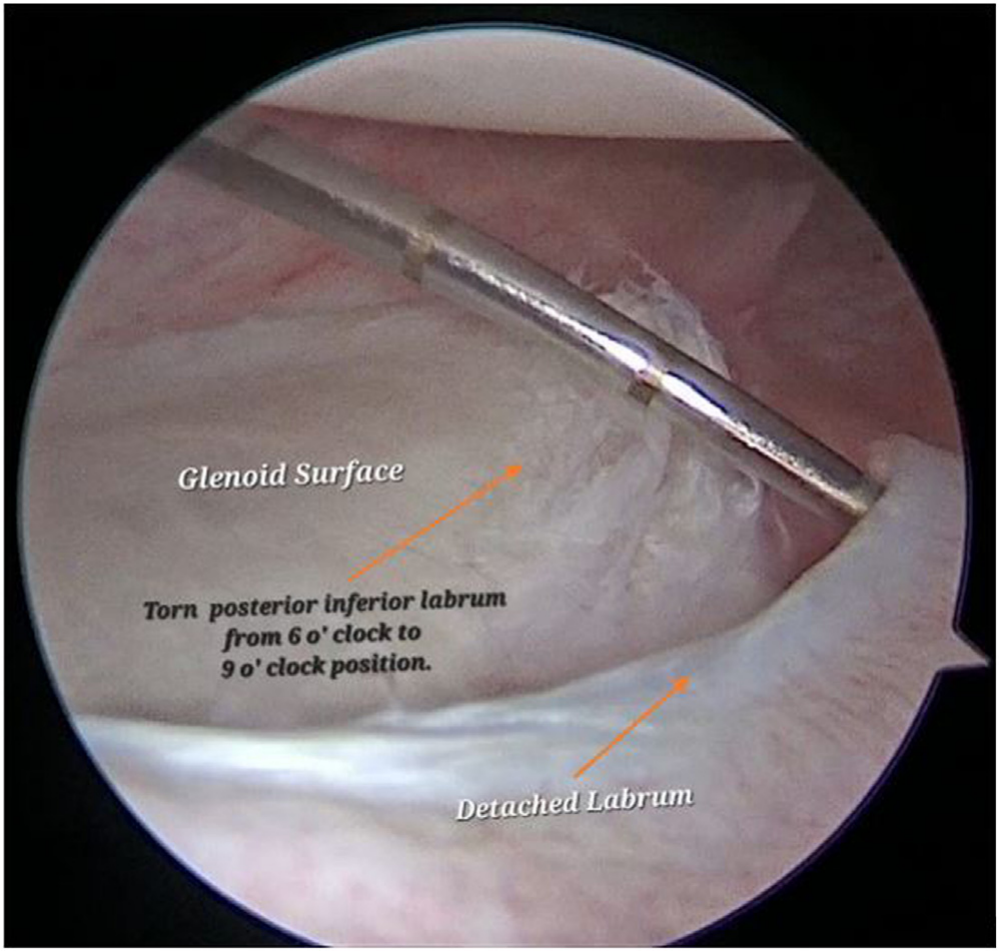
With patient in lateral decubitus, performing a diagnostic arthroscopy from posterior portal and probing from the antero-superior portal, a torn and separated posterior-inferior labrum from the glenoid at 6-o’ clock to 9-o’ clock position is identified.

**Fig 2:**
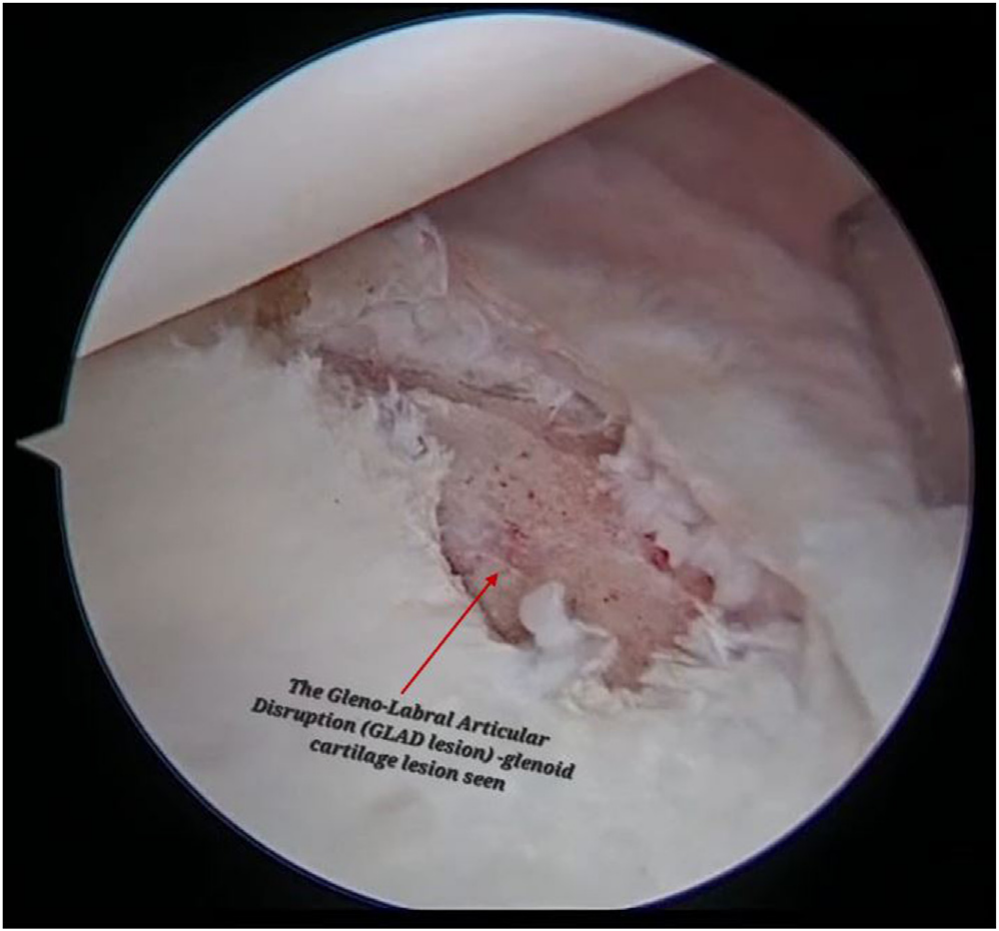
Diagnostic arthroscopy of the right shoulder through the posterior viewing portal with patient in lateral position, shows a glenolabral articular disruption (GLAD) lesion): cartilage defect is see on the face of the glenoid.

Diagnostic arthroscopy with 30° arthroscope is performed after creating standard posterior, anteroinferior, and anterosuperior portals (Video 1). In this patient, the posteroinferior labrum was torn and separated from the glenoid from the 6- to 9-o’clock position (Fig 1, Video 1). A cartilage defect was identified on the glenoid from the 6- to 9-o’clock position (Fig 2, Video 1). The defect was found to be covered with fibrous tissue.

**Fig 3:**
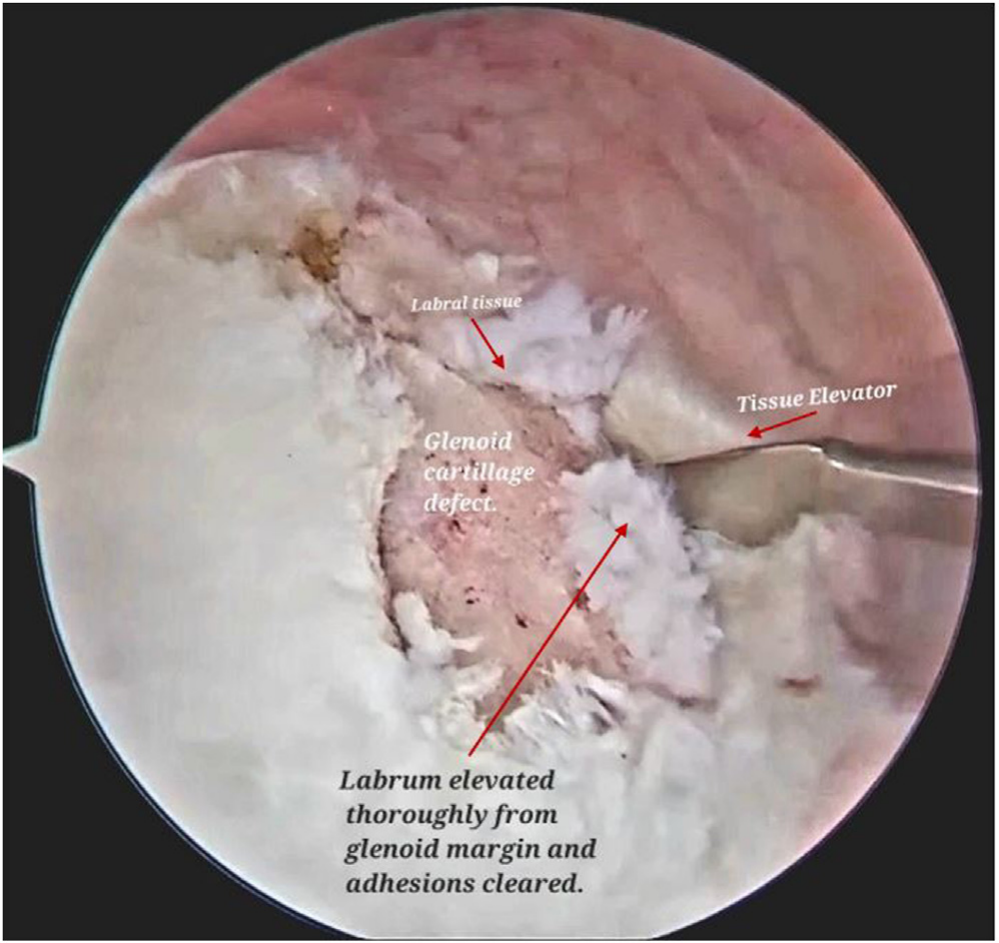
Arthroscopic view of right shoulder with the patient in lateral position and visualizing from posterior viewing portal, labrum tissue is elevated with the help of a tissue elevator, introduced from the anteroinferior working portal, until all adhesions are cleared and the underlying muscle is visualized.

Visualizing from the posterior portal, we elevate the labrum tissue with the help of a tissue elevator, introduced from the anteroinferior portal, until all adhesions are cleared and the underlying muscle is visualized (Fig 3, Video 1). Loose fibrous tissue is removed with a motorized shaver system.

**Fig 4:**
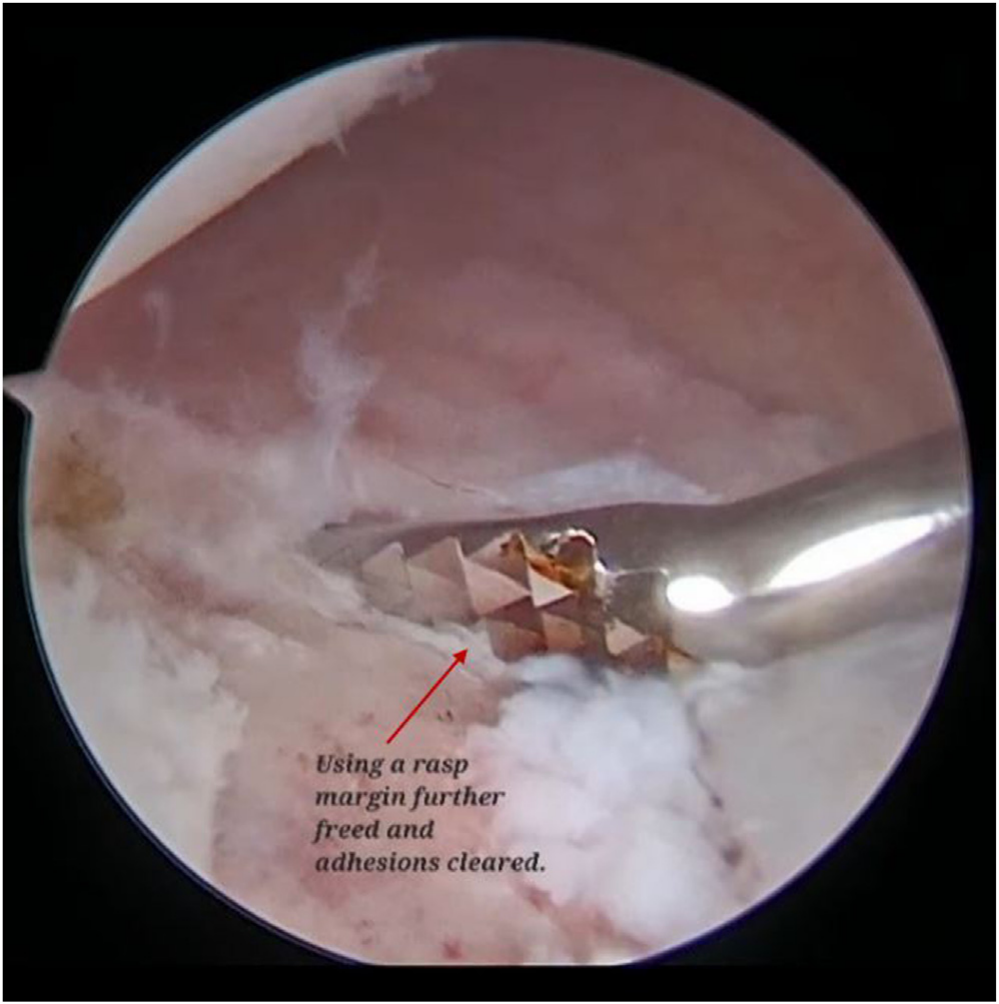
Arthroscopic view of right shoulder with the patient in lateral position with the scope is switched to the anterosuperior viewing portal, and a tissue liberator and then rasp are passed through the posterior working portal. Adhesions are further cleared, glenoid margin is abraded, and the bone is prepared.

The scope is then shifted to the anterosuperior portal. An 8.25-mm cannula (Twist-In Cannula, 8.25 mm I.D. _ 9 cm; Arthrex) is pushed through the posterior portal. Now we shift the scope to the anterosuperior portal, passing a tissue liberator and then a rasp through the posterior working portal. Adhesions are further cleared, the glenoid margin is abraded, and the bone is prepared (Fig 4).

**Fig 5:**
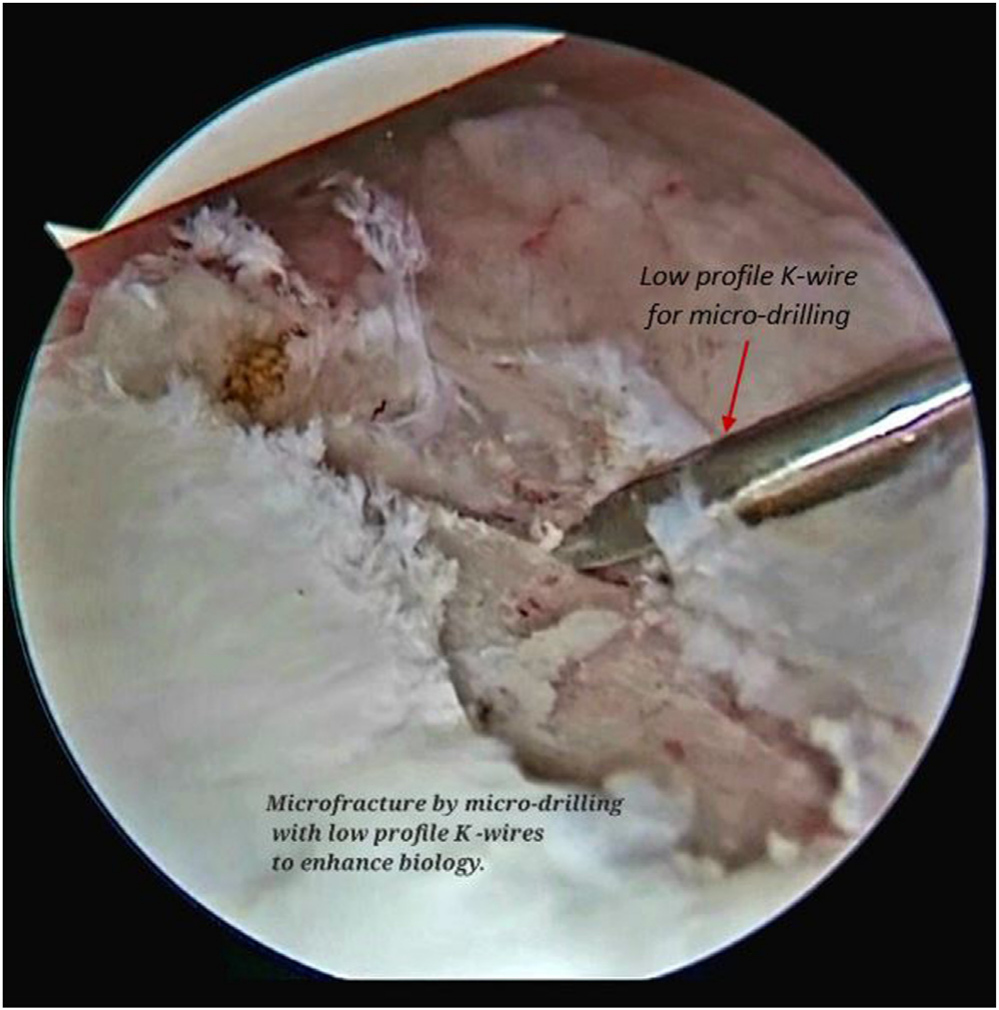
Arthroscopic view of right shoulder with the patient in lateral position and the scope in the anterosuperior viewing portal and microfracture and multiple micro-drilling on the lesion is performed with low-profile K-wires using the posterior working portal to promote bleeding and enhance the biology for better tissue healing.

**Fig 6:**
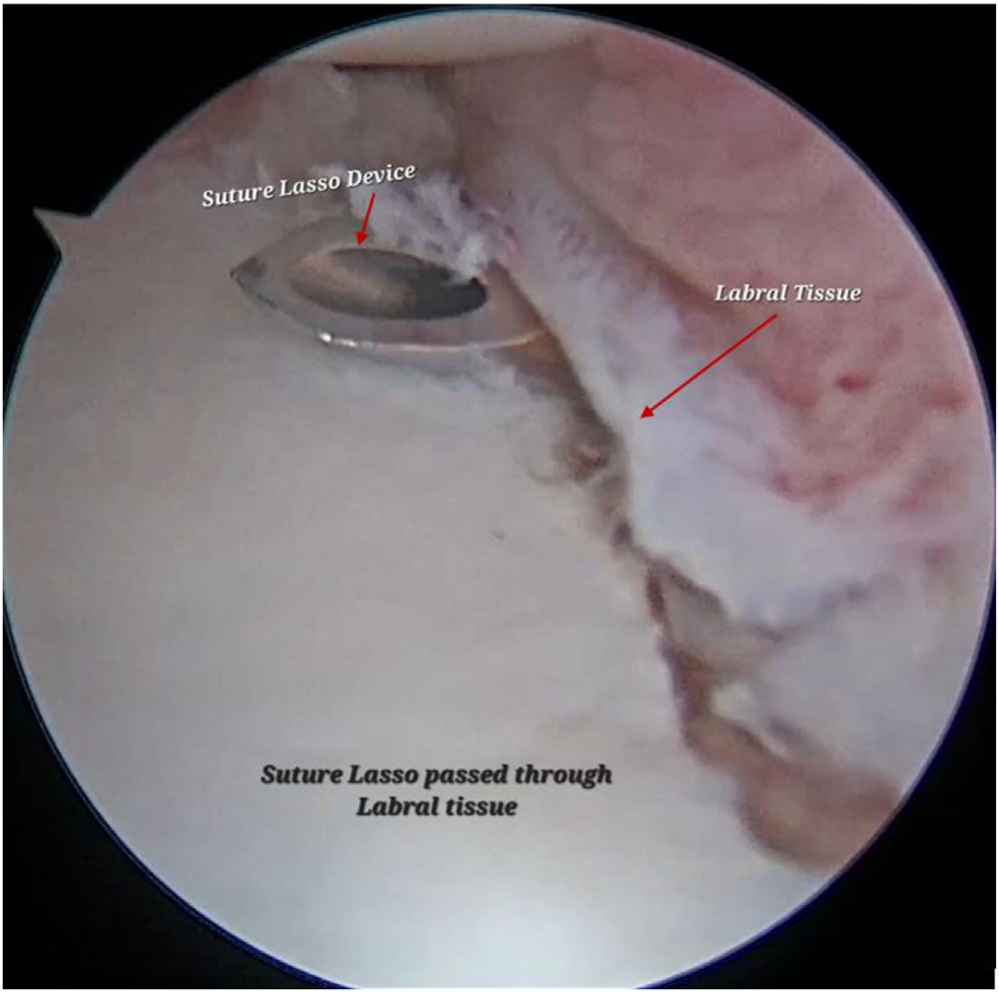
Arthroscopic view of right shoulder through the antero-superior portal with the patient in lateral position showing a SutureLasso (Arthrex SutureLasso SD 25-degree tight curve left) being passed through the labrum and adjacent capsule from the posterior working portal.

Microfracture and multiple microdrilling is performed with low profile K-wires to promote bleeding and enhance the biology for better tissue healing (Fig 5, Video 1). After that, a SutureLasso (SutureLasso SD 25-degree tight curve left; Arthrex) is passed through the labrum and adjacent capsule (Fig 6, Video 1).

**Fig 7:**
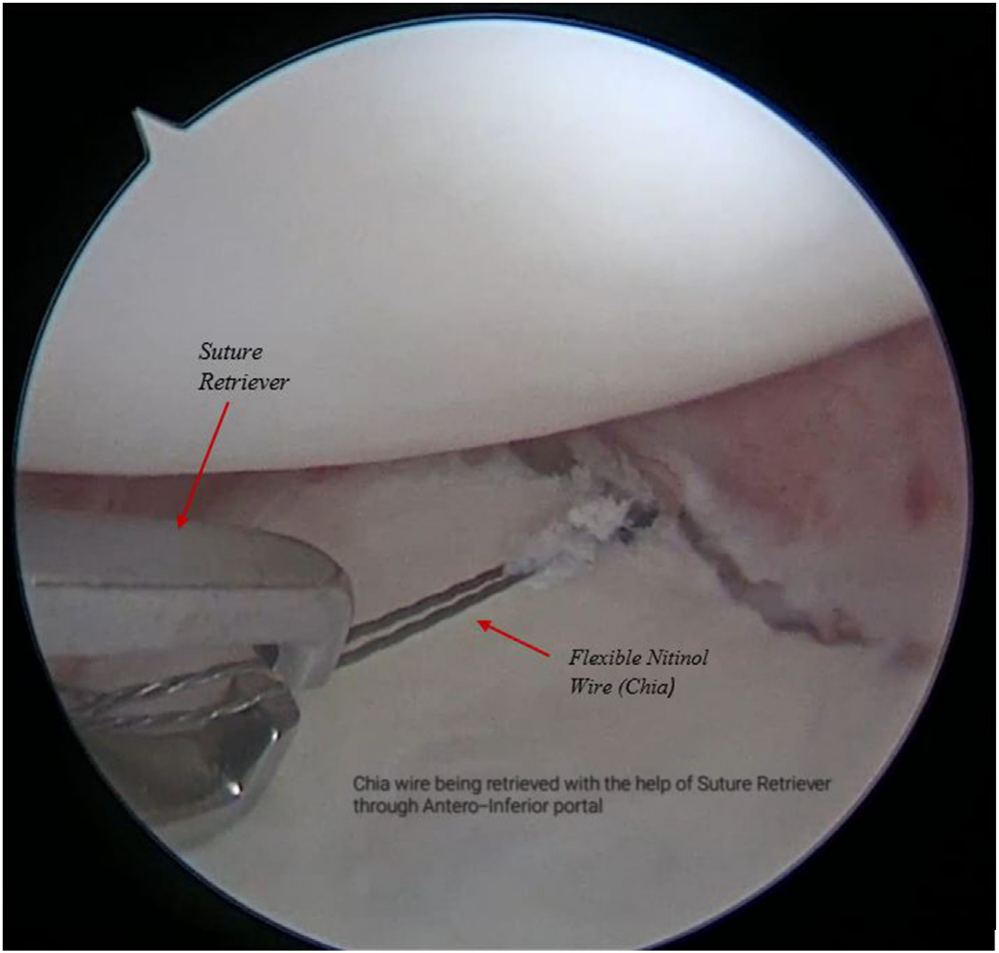
Arthroscopic view of right shoulder from the antero-superior viewing portal with patient in lateral position showing a flexible, twisted nitinol wire being delivered using the SutureLasso from the posterior portal, across the labrum and is retrieved through antero-inferior portal.

A flexible twisted nitinol wire is delivered through the lasso and retrieved through the anteroinferior portal using a suture retriever (Fig 7, Video 1).

**Fig 8:**
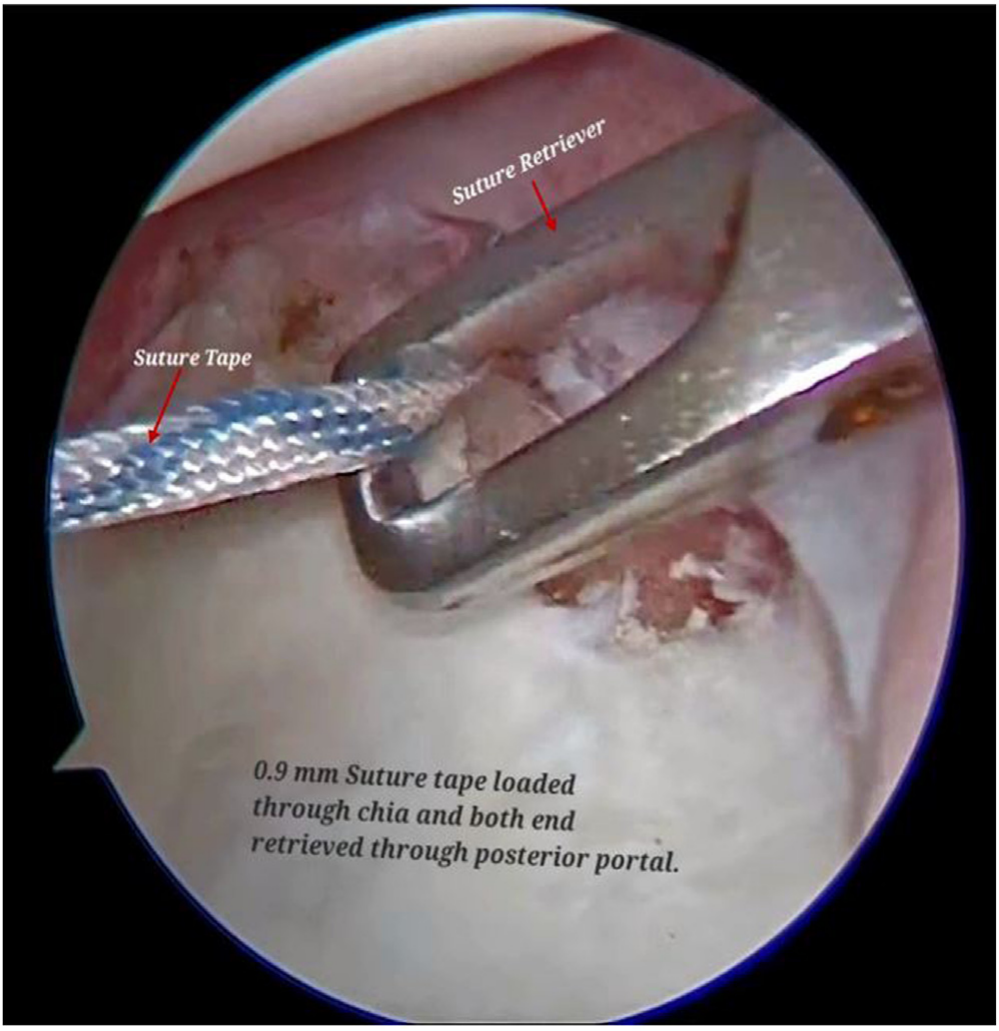
Arthroscopic view of right shoulder from the antero-superior viewing portal with patient in lateral position showing a low-profile 0.9-mm microsuture tape being loaded over the flexible twisted nitinol wire, brought from the antero-inferior portal, and retrieved through the labrum and capsule into the posterior working portal.

A low-profile 0.9-mm suture tape (SutureTape; Arthrex) is loaded over the flexible twisted nitinol wire, which is retrieved through the labrum and capsule into posterior portal. The other end of SutureTape is also retrieved through posterior portal (Fig 8, Video 1).

**Fig 9:**
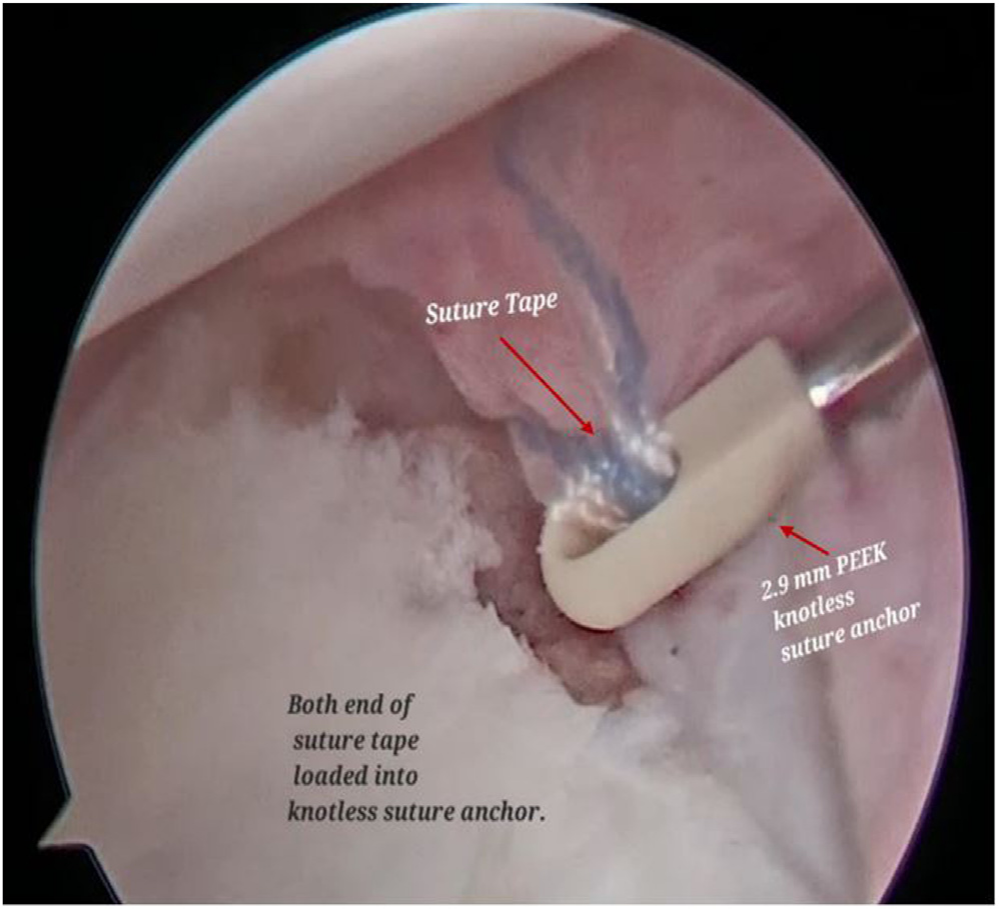
Arthroscopic view of right shoulder from the antero-superior viewing portal with patient in lateral position showing both ends of the SutureTape being loaded on a 2.9-mm PEEK knotless suture anchor (PushLock, Arthrex), and introduced through the posterior working portal.

**Fig 10:**
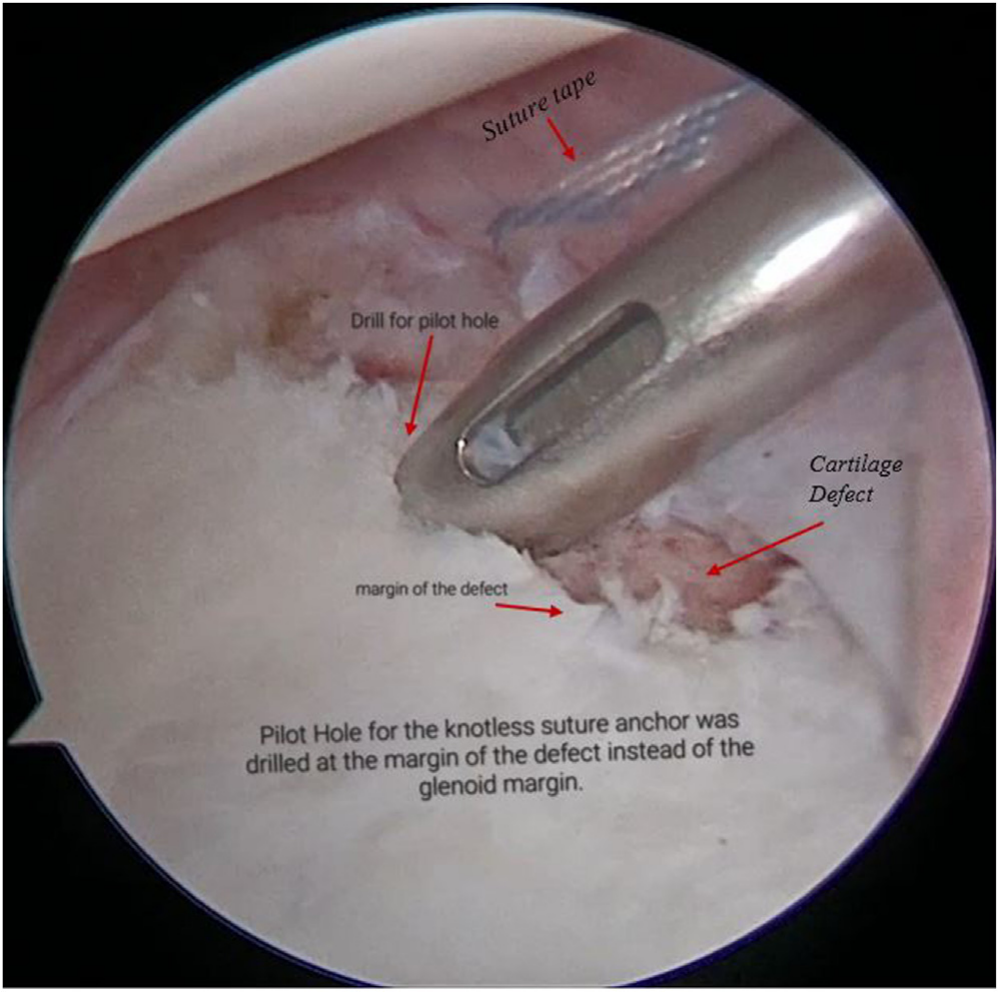
Now viewing the right shoulder of the patient in lateral position using the antero-superior viewing portal and using the posterior working portal, a pilot hole for the knotless push-lock anchor is made at the margin of the defect instead of the glenoid margin.

**Fig 11:**
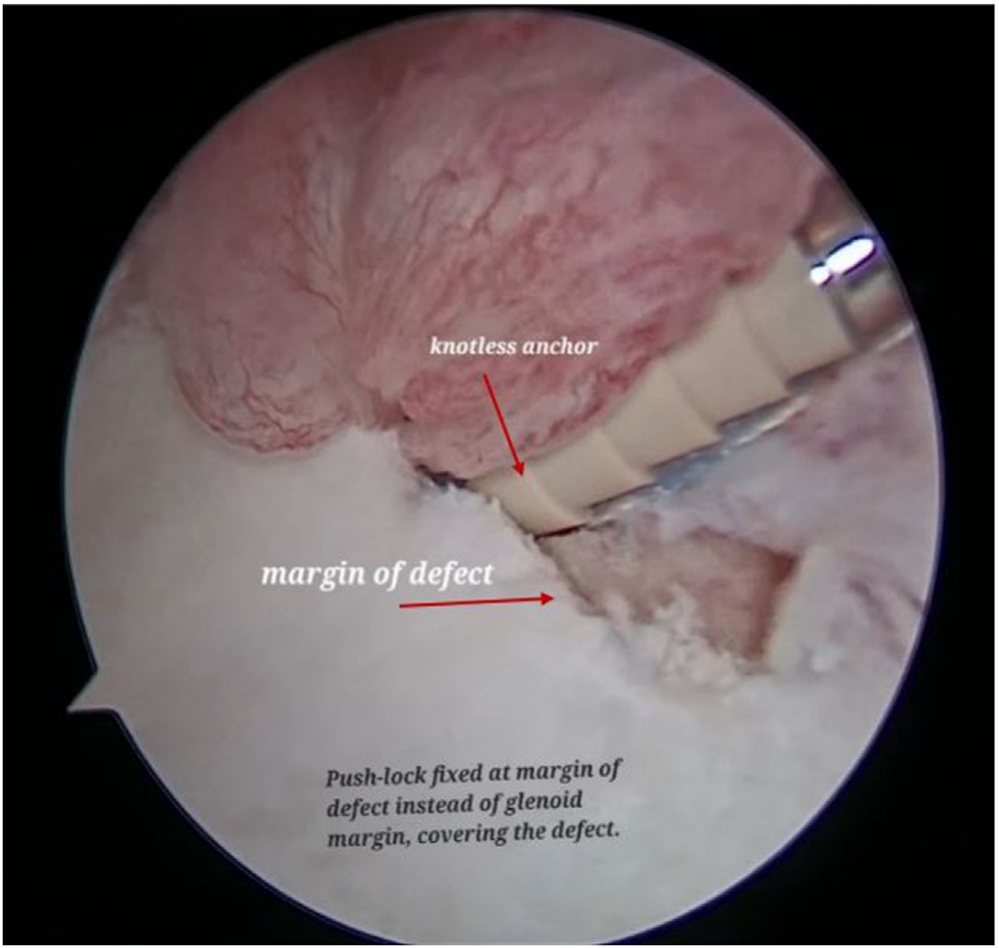
Now visualising from the antero-superior portal, after appropriate size drilling of the pilot hole using the posterior portal, the PushLock anchor with the SutureTape is fixed on the margin of the defect.

**Fig 12:**
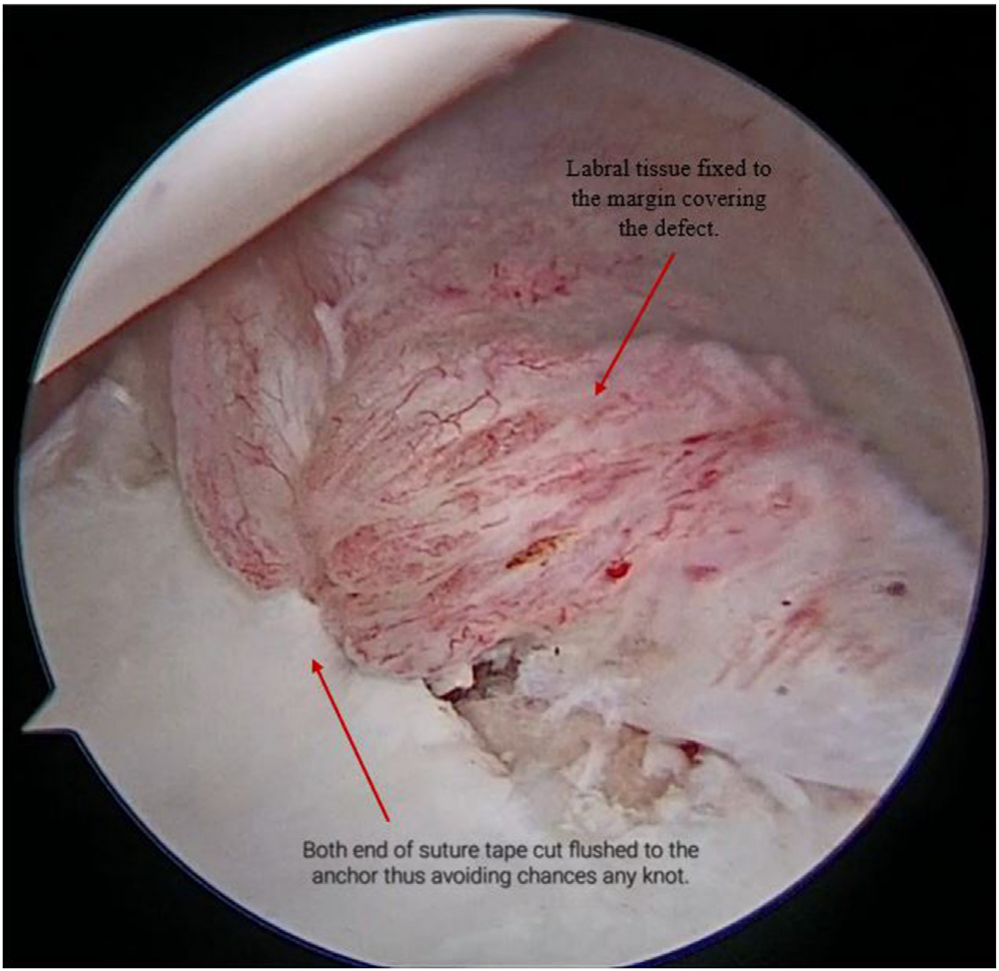
Now viewing the right shoulder of the patient in lateral decubitus position using the antero-superior viewing portal and after securing the knots, both ends of the defect are cut flush to the anchor with suture cutter, thereby leaving no knot around anchor or soft tissue.

Both the ends of SutureTape are loaded on a 2.9-mm PEEK (polyether ether ketone)knotless suture anchor (PushLock; Arthrex) (Fig 9, Video 1). After appropriate size drilling of pilot hole, the PushLock anchor is placed on the margin of the defect instead of glenoid margin (Fig 10, Video 1). Before the anchor is pushed in, both ends of the suture tape are pulled. As the anchor is driven in, the capsulolabral complex advances as a result and becomes attached to the margin of defect (Fig 11, Video 1). Both ends of the tape are cut flush to anchor with suture cutter (Arthrex), thereby leaving a knotless configuration with no knot around anchor or in soft tissue preventing any excoriation on the glenoid or humeral head cartilage (Fig 12).

**Fig 13:**
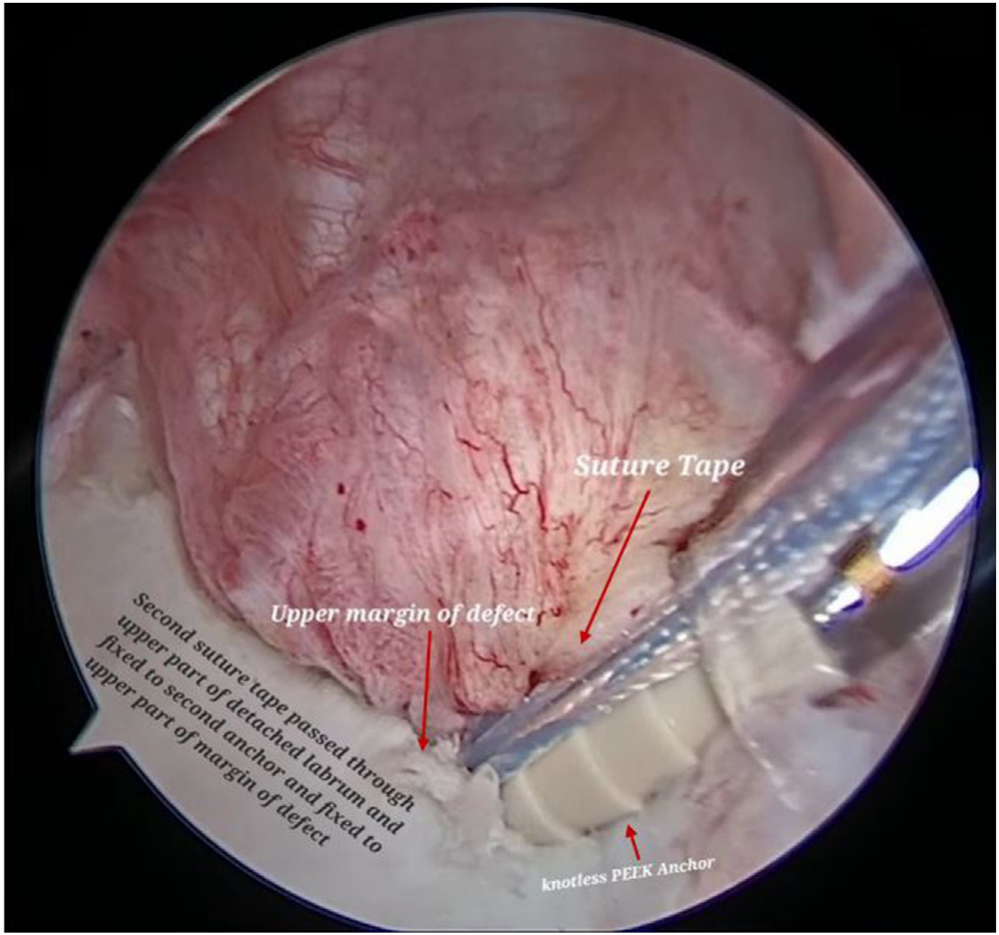
Now, using the same viewing portal and working portal, on right shoulder of the patient in lateral decubitus position, a second SutureTape and second PushLock anchor are inserted to fix proximal part of margin of defect.

**Fig 14:**
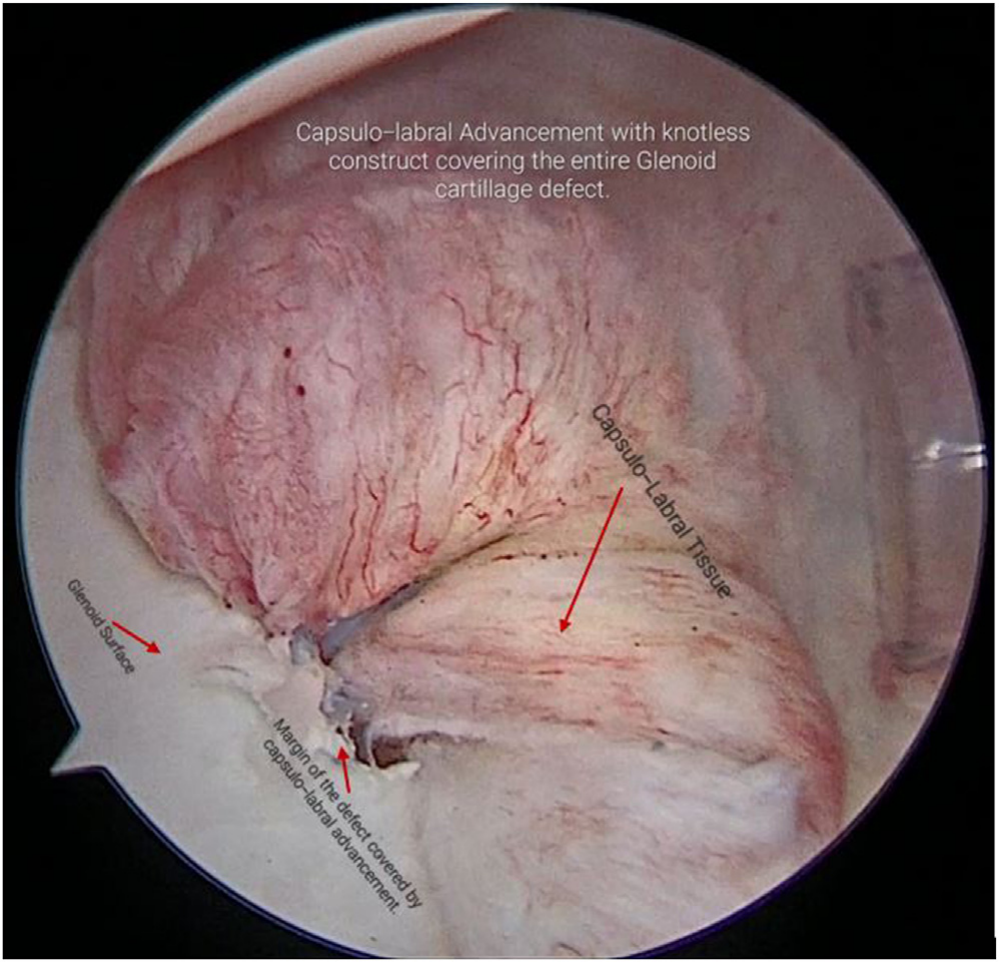
Arthroscopic view of right shoulder with patient in lateral decubitus position showing final stable knotless construct with capsulo-labral advancement covering entire glenoid cartilage defect as is viewed from the anteroinferior portal.

A second SutureTape is passed through proximal part of detached labrum and adjacent capsule and both ends of SutureTape loaded to a second PushLock anchor (2.9-mm PEEK; Arthrex), which is fixed to the proximal part of margin of defect (Fig 13, Video 1). After capsulolabral advancement and fixation, the entire cartilage defect is covered, without leaving any knots that can abrade the cartilage (Fig 14, Video 1).

Finally, all portals are closed, a dressing is applied, and the shoulder immobilized in neutral rotation with appropriate size shoulder immobiliser.

## Discussion

Several surgical strategies have been reported for glenoid articular cartilage defects, which includes debridement, microfracture, osteochondral grafting, and autologous chondrocyte implantation. In a case report, Agarwal et al.^9^ described managing GLAD lesions with combined repair of the labral flap with labral tear. They used glenoid rim anchors with suture passed through the fibrous rim of the displaced cartilage flap. Pogorzelski et al.^10^ found GLAD lesions increased the risk of failure after arthroscopic Bankart repair. They managed GLAD lesions with debridement of unstable flaps then microfracture and advancement of the labrum into the defect.

Our technique of capsulolabral advancement and “suture-first” knot-less technique for posterior GLAD lesion of the shoulder has several advantages (Table 1). The use of knotless anchors avoids bulky knots, as seen with knotted anchors, and decreases cartilage erosion. Our technique showcases microfracture of defect by microdrilling with low-profile K-wires, which enhances biology, improving healing (Table 1). It is important to remove unstable chondral flaps to achieve stable contained borders before microfracture (Table 2). In our technique, fixation of the labrum at the margin of the defect covers the exposed subchondral glenoid area with capsulolabral tissue and decreases the chance of ensuing arthritis. The main concern regarding our technique that because we are advancing the capsulolabral tissue anteriorly, there may be postoperative stiffness on posterior aspect of shoulder with potential for loss of range of motion (Table 2).

**Table 1:** Advantages and Disadvantages of Our Technique of Arthroscopic Posterior Glenolabral Articular Disruption (GLAD) Lesion Management Using Knotless Anchors

Advantages	Disadvantages
• Microfracture in the form of microdrilling with low-profile K-wires of defect accelerates biological healing.	• Repair of chronic GLAD lesion is more challenging compared with acute lesions.
• Removal of fibrous tissue over cartilage defect using a shaver system to expose raw area also enhances biological healing of the tissues.	• Overadvancement of capsulolabral tissue may tighten shoulder capsule and worsen range of motion.
• Use of knotless anchors reduce chances of abrasion and scuffing of cartilage.	• In case of large glenoid chondral defects, advancement of capsulolabral tissue over the defect is sometimes inadequate and fails to cover the entire lesion.

**Table 2:** Pearls and Pitfalls of Capsulolabral Advancement and “Suture-First” Knot-Less Technique for Chronic Posterior Glenolabral Articular Disruption (GLAD) Lesion

Pearls	Pitfalls
• Placing anchors on the medial margin of articular defect and advancing capsulolabral tissue onto lesion covers the lesion, reducing exposed subchondral bone.	• Proper liberation of the labral tissue should be done as inadequate tissue elevation, will limit its excursion onto the defect.
• Before driving the anchor, both ends of suture tape are pulled and we subsequently drive-in the suture, leading to the advancement of the capsulolabral complex and attachment to the margin of the defect.	• Placement of anchors more medial than possible labrum tissue advancement will result in a weakened construct.
• Delineating the medial extension of the capsulolabral tissue before placing anchors should be done to ensure appropriate placement of anchors with full coverage of defect.	
• Unstable cartilage flaps should be debrided and defect’s border should be defined before capsulolabral advancement.	

To summarize, management of GLAD lesions consist of restoring capsulolabral integrity, debriding loose cartilage flaps, promoting fibrocartilage formation through microfracture, and advancement of the capsulolabrum complex.^11^^,^^12^ Our technique, while encompassing these principles to treat a chronic posterior GLAD lesion, also showcases the advantages of using “suture-first” knotless technique, thus preventing abrasion and scuffing of humeral head cartilage.

## Disclosures

All authors (R.P., A.S.) declare that they have no known competing financial interests or personal relationships that could have appeared to influence the work reported in this paper.

## Supplementary Data

Video 1 Capsulo-labral advancement and “suture-first” knot-less technique for chronic posterior Gleno-labral articular disruption (GLAD) lesion of right shoulder with patient in lateral position. Viewing and working portals have been mentioned in the video accordingly with the steps of the procedure.

## References

[1] Neviaser T.J. (1993). The GLAD lesion: Another cause of anterior shoulder pain. Arthroscopy.

[2] Page R., Bhatia D.N. (2010). Arthroscopic repair of a chondro-labral lesion associated with anterior glenohumeral dislocation. Knee Surg Sports Traumatol Arthrosc.

[3] Antonio G.E., Griffith J.F., Yu A.B., Yung P.S.H., Chan K.M., Ahuja A.T. (2007). First-time shoulder dislocation: High prevalence of labral injury and age-related differences revealed by MR arthrography. J Magn Reson Imaging.

[4] Zhu W., Lu W., Zhang L. (2014). Arthroscopic findings in the recurrent anterior instability of the shoulder. Eur J Orthop Surg Traumatol.

[5] Ruckstuhl H., de Bruin E.D., Stussi E., Vanwanseele B. (2008). Posttraumatic glenohumeral cartilage lesions: A systematic review. BMC Musculoskelet Disord.

[6] Knight J.A., Powell G.M., Johnson A.C. (2024). Radiographic and advanced imaging evaluation of posterior shoulder instability. Curr Rev Musculoskelet Med.

[7] Siebold R., Lichtenberg S., Habermeyer P. (2003). Combination of microfracture and periostal-flap for the treatment of focal full thickness articular cartilage lesions of the shoulder: A prospective study. Knee Surg Sports Traumatol Arthrosc.

[8] Porcellini G., Cecere A.B., Giorgini A., Micheloni G.M., Tarallo L. (2020). The GLAD lesion: Are the definition, diagnosis and treatment up to date? A systematic review. Acta Biomed.

[9] Agarwalla A., Puzzitiello R.N., Leong N.L., Forsythe B. (2019). Concurrent primary repair of a glenoid labrum articular disruption and a Bankart lesion in an adolescent: A case report of a novel technique. Case Rep Orthop.

[10] Pogorzelski J., Fritz E.M., Horan M.P., Katthagen J.C., Provencher M.T., Millett P.J. (2018). Failure following arthroscopic Bankart repair for traumatic anteroinferior instability of the shoulder: Was a glenoid labral articular disruption (GLAD) lesion a risk factor for recurrent instability?. J Shoulder Elbow Surg.

[11] Do A., Pieringer A., Scheibel M. (2024). Surgical treatment of a glenolabral articular disruption lesion using knotless labral repair and minced cartilage procedure: A case report. JSES Rev Rep Tech.

[12] Lin S., Zhong Z., Xiao J. (2024). A new, effective method for diagnosing GLAD lesions: The chicken-wing muscle up test. BMC Musculoskelet Disord.

